# U-shaped and L-shaped Left Main Coronary Artery: Rare Congenital Anomalies

**DOI:** 10.7759/cureus.47131

**Published:** 2023-10-16

**Authors:** Dibyasundar Mahanta, Debasis Acharya, Debasish Das

**Affiliations:** 1 Department of Cardiology, SUM Hospital, Bhubaneswar, IND; 2 Department of Cardiology, All India Institute of Medical Sciences, Bhubaneswar, Bhubaneswar, IND

**Keywords:** l-shape, u-shape, congenital anomaly, rare, left main coronary artery, alphabetical

## Abstract

Congenital anomalies in the shape of the coronary arteries are extremely uncommon to encounter in routine clinical practice in interventional cardiology. In this study, we describe two uncommon shape anomalies of the left main coronary artery, that is, U-shaped left main coronary artery and L-shaped left main coronary artery. These anomalies were observed in two consecutive patients who presented with atypical chest pain and exertional shortness of breath. These uncommon shape anomalies of the left main coronary artery hold their clinical significance during intervention as those require robust guide catheter support during the difficult passage of the routine coronary hardwires.

## Introduction

Coronary anomalies affect 1% of the general population when invasive coronary angiogram is taken into account [1-3]. There is not much description of shape anomalies in the coronary arteries. However, those shape anomalies are of paramount importance during the coronary intervention as it is difficult to pass the routine coronary hardwires when there is an associated shape anomaly. Shape anomalies in the coronary arteries may predispose to shear stress during coronary blood flow, which may manifest as exercise-limiting angina and shortness of breath. Acute angle takeoff of coronary arteries from the coronary sinus can also result in exercise-induced ischemia in the individuals. We report two incidentally detected rare congenital shape anomalies of the left main coronary artery: an inverted U-shape and an L-shape. These anomalies were likely the culprits behind exercise-induced angina, in addition to traditional atherosclerosis risk factors. Robust guide catheter support is required in such coronaries with anomalous shapes to provide smooth passage of routine coronary hardwires during coronary intervention. 

## Case presentation

Case 1

**Figure 1:**
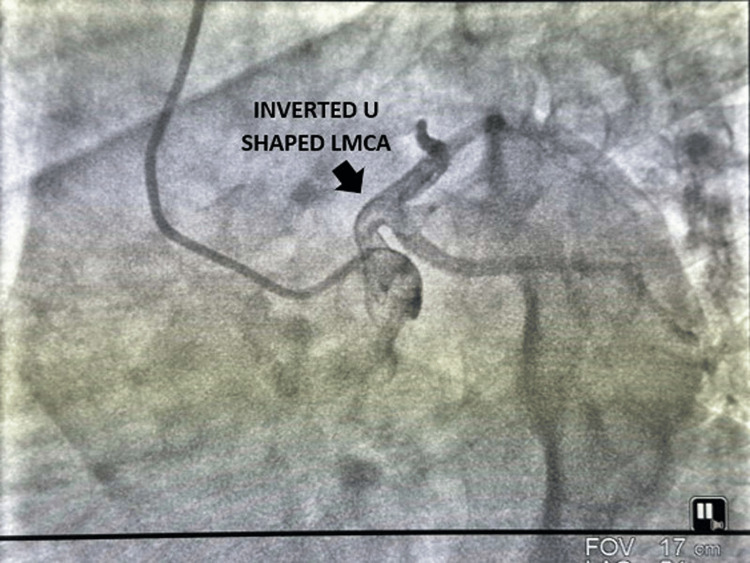
Inverted U-shaped left main coronary artery (LMCA) in LAO caudal view (spider view). LAO, left anterior oblique

A 56-year-old male, diabetic, hypertensive, and a smoker, presented to the outpatient department (OPD) with effort intolerance, categorized as New York Heart Association (NYHA) Class II, and reported diaphoresis over the last six months, without any history of palpitation, presyncope, or syncope. His heart rate was 80 beats per minute, and his blood pressure was 150/90 mmHg in the right arm supine position. His electrocardiogram (ECG) was within normal limits, and his echocardiography revealed no regional wall motion abnormality with normal ejection fraction. His exercise treadmill test was borderline positive, and he was subjected to an invasive coronary angiogram due to effort-limiting angina. A coronary angiogram revealed an inverted U-shaped left main coronary artery (Figure 1), normally bifurcating into a left anterior descending coronary artery (LAD) and left circumflex coronary artery (LCX) without any luminal obstruction with a normal right coronary artery. Increased tortuosity in the form of an inverted U-shape increases shear stress during coronary blood flow, which may be the contributing factor toward the development of effort angina in the aforesaid patient.

Case 2

**Figure 2:**
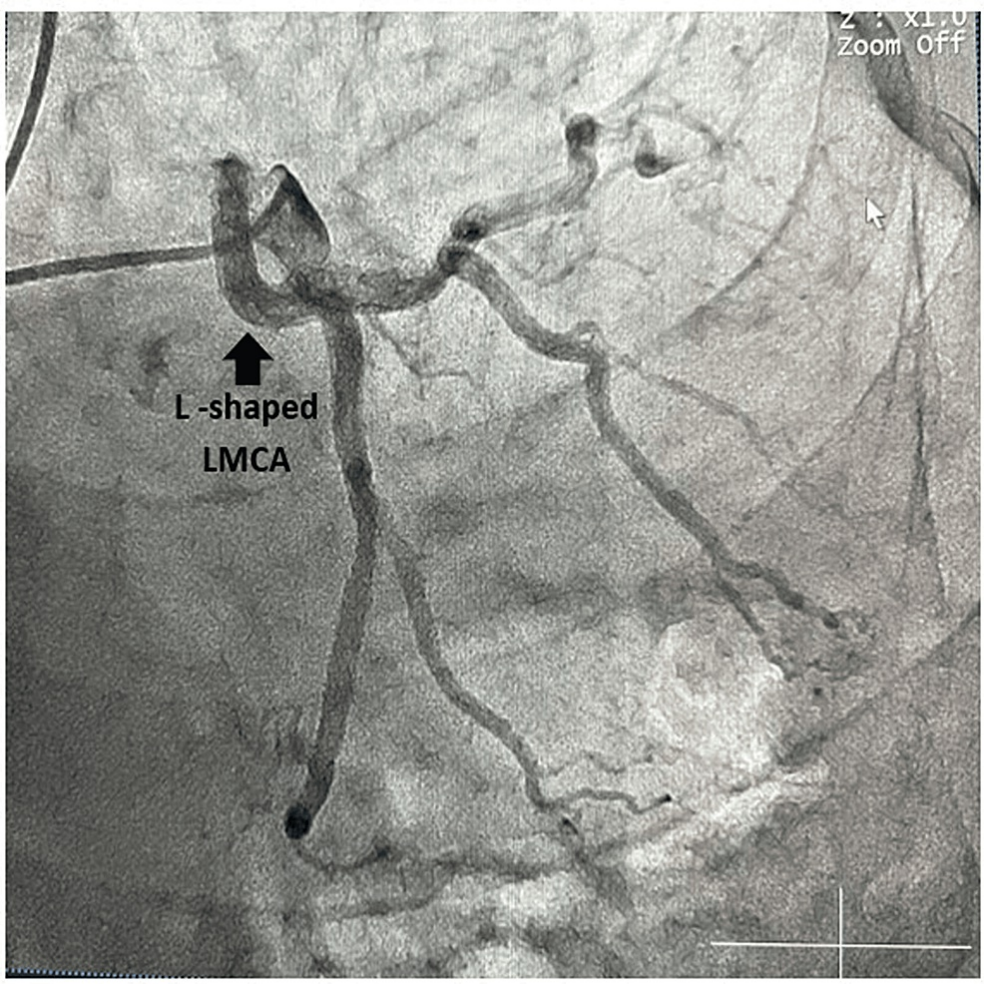
L-shaped left main coronary artery (LMCA) in LAO caudal view (spider view). LAO, left anterior oblique

A 62-year-old male diabetic, hypertensive, and obese presented to the cardiology OPD with effort angina Canadian Cardiovascular System (CCS) Classification II for the last six months. He had a heart rate of 80 beats per minute and blood pressure of 144/80 mmHg in the right arm supine position. His ECG and echocardiography were within normal limits. He could not perform an exercise treadmill test due to the presence of osteoarthritis. He was on single antiplatelet therapy, statin, beta-blocker, and nitrate for the last six months. As his angina was not responsive to optimal medical therapy (OMT), he was subjected to a coronary angiogram. An invasive coronary angiogram revealed an L-shaped anomaly of the left coronary artery (Figure 2) without any atherosclerotic luminal obstruction in any of the coronaries. The presence of an acute 90-degree bend in the coronary artery, forming an L-shape, increased shear stress in the coronary blood flow during exertion. This contributed to the development of effort angina in the aforementioned patient. Additionally, a close differential diagnosis included microvascular angina due to the dual presence of diabetes and obesity, further increasing the likelihood of microvascular obstruction leading to angina.

## Discussion

The incidence of coronary anomalies in routine echocardiography is estimated to be around 0.17% [4], while the incidence of coronary anomalies during coronary angiography is estimated to be about 1.07% [5]. In Angelini's classic series of coronary anomalies, shape anomalies of the coronaries are not much described. We describe uncommon shape anomalies of coronary arteries, that is, U-shaped and L-shaped anomalies of the left main coronary arteries in patients with exertional angina. Careful examination of the course of the coronary artery is important as an anomalous coronary artery running between the aorta and the pulmonary artery carries the highest risk of mortality due to ventricular arrhythmias and risk of sudden cardiac death [6]. These U-shaped anomalies with two 90-degree bends and L-shaped anomalies with a single 90-degree bend in the proximal part of the coronaries may increase the shear stress during coronary blood flow, especially during exertion, which may contribute toward effort angina in those patients. Arteries with atherosclerotic lesions exhibiting bends and tortuosities are categorized as type B and type C coronary lesions. These types can pose challenges during interventions, particularly when navigating hardwires. During the intervention of those types of coronary anomalies, the use of a larger guide catheter, the use of intermediate strength or extra support guidewires, and the use of parallel guide wire technique can overcome the difficulty in passing the balloon or the coronary stent across the 90-degree turns. The use of slippery or hydrophilic guidewires eases crossing those bends in U- or L-shaped coronaries as compared to the use of nonhydrophilic guidewires. Sometimes, it becomes difficult to even engage the coronary artery with proximal shape anomaly; nonselective hand injection of the sinus with 10-20 mL of contrast can opacify such proximal anomalies and delineate the coronary lesions.

## Conclusions

We describe the rarest shape anomalies of the LMCA, that is, U-shaped LMCA and L-shaped LMCA, in patients with exertional angina, which have not been described in the literature so far. Although these anomalies are uncommon to be encountered during routine interventional practice, they exert their importance during exertion by increasing the shear stress in coronary blood flow and producing relative coronary ischemia. Shape anomalies of the coronary arteries also carry their importance during the intervention as those acute turns pose a great challenge while passing the routine coronary hardwires.
